# Gas Poisoning Hints

**Published:** 1891-04

**Authors:** 


### GAS POISONING HINTS.

Loosen the clothing at the neck.

Slap the face and chest with the wet end of a towel.

Apply warmth and friction if the body or limbs are cold.

Take the man at once into the fresh air. Don't crowd around him.

Keep him on his back. Don't raise his head or turn him on his side.

If the breathing is feeble or irregular artificial respiration should be used and kept up until there is no doubt that it can no longer be of use.

Give the ammonia mixture (one part in all, aromatic ammonia, to sixteen parts of water) in small quantities at short intervals, a teaspoonful every two or three minutes.

